# Italian Contributions to the Development of Continuous Glucose Monitoring Sensors for Diabetes Management

**DOI:** 10.3390/s121013753

**Published:** 2012-10-12

**Authors:** Giovanni Sparacino, Mattia Zanon, Andrea Facchinetti, Chiara Zecchin, Alberto Maran, Claudio Cobelli

**Affiliations:** 1 Department of Information Engineering, University of Padova, Via Gradenigo 6/B, 35131 Padova, Italy; E-Mails: gianni@dei.unipd.it (G.S.); zanonma1@dei.unipd.it (M.Z.); facchine@dei.unipd.it (A.F.); zecchinc@dei.unipd.it (C.Z.); 2 Department of Clinical and Experimental Medicine, Metabolic Division, University of Padova, Via Giustiniani 2, 35128 Padova, Italy; E-Mail: alberto.maran@unipd.it

**Keywords:** diabetes, biosensors, non-invasive sensors, glucose-oxidase, signal processing, sensor calibration, model, time-series

## Abstract

Monitoring glucose concentration in the blood is essential in the therapy of diabetes, a pathology which affects about 350 million people around the World (three million in Italy), causes more than four million deaths per year and consumes a significant portion of the budget of national health systems (10% in Italy). In the last 15 years, several sensors with different degree of invasiveness have been proposed to monitor glycemia in a quasi-continuous way (up to 1 sample/min rate) for relatively long intervals (up to 7 consecutive days). These continuous glucose monitoring (CGM) sensors have opened new scenarios to assess, off-line, the effectiveness of individual patient therapeutic plans from the retrospective analysis of glucose time-series, but have also stimulated the development of innovative on-line applications, such as hypo/hyper-glycemia alert systems and artificial pancreas closed-loop control algorithms. In this review, we illustrate some significant Italian contributions, both from industry and academia, to the growth of the CGM sensors research area. In particular, technological, algorithmic and clinical developments performed in Italy will be discussed and put in relation with the advances obtained in the field in the wider international research community.

## 1. Introduction

Glucose represents the most important fuel for human beings and its level in the blood is precisely controlled by insulin. In diabetic patients, the pancreas does not secrete insulin (type 1 diabetes) or problems in both insulin secretion and action (type 2 diabetes) occur, and as a consequence, glucose concentrations in blood often exceed the normal range (70–180 mg/dL). Hyperglycemia mostly creates serious long-term complications, such as neuropathy, retinopathy and cardiovascular and heart diseases, while hypoglycemia is of dramatic concern in the short-term, since it can turn into dangerous episodes, e.g., hypoglycemic coma, especially at night when the patient is asleep.

About 350 million people worldwide are estimated to be diabetic (50% of whom are undiagnosed) [1]. In Italy, an ISTAT survey in 2010 estimated three million diabetic citizens [2]. Worldwide, the number of patients is rapidly increasing due to aging populations, sedentary lifestyles and increasing obesity [3], with the prospective of exceeding 500 million cases in 2030. Diabetes and its complications are considered major causes of early death in most countries, with over four million deaths per year [1]. From an economic point of view, the cost of diabetes ranges from 6 to 15% of the budget of national health systems in the EU, e.g., in 2011, Italy spent about 10 billion Euros for diabetes [4,5]. This explains why diabetes is considered one of the most challenging socio-health emergencies of the 3rd millennium [6] and also why the impact of innovative methodologies and technologies for diabetes monitoring and treatment developed by researchers from both academia and commercial companies can be extremely high, with a potential market of several billions of Euros.

Several clinical studies have demonstrated that the long-term diabetes complications, due to hyperglycemia, and the frequency of risky short-term complications, due to hypoglycemia, could be reduced through a therapy based on a mix of diet, physical exercise, and drug delivery (including subcutaneous injections of exogenous insulin), tuned according to the monitoring of individual parameters [1]. This has stimulated the development of a plethora of methods, based on models and algorithms with different degree of sophistication, for efficient monitoring and management of diabetes [7]. Most of these models and algorithms rely on the capability of correctly measuring glucose concentration levels in the blood by using suitable sensors.

In the most commonly used approach, the diabetic patient self-monitors his glycemia 3–4 times per day, by using portable minimally-invasive lancing sensor devices [8], which exploit the glucose-oxidase enzyme and return measurements called self-monitoring blood glucose (SMBG). In the majority of cases, SMBG time-series are collected by the patient and then analyzed and interpreted by the physician during periodic visits, e.g., every two/four months. Then, the individual therapeutic plan is revised accordingly. The automation of some of these processes and the introduction of systems based on data analysis to support clinical decisions have been studied since the beginning of the 90s and several telemedicine infrastructures have been proposed, see e.g., [9] and references quoted therein, together with algorithms to analyze SMBG data and their variability, e.g., [10–13]. SMBG samples can also be used in real-time by the patient to assess somewhat the current effectiveness of glucose control, but due to their sparseness, they cannot give a complete information of glycemic excursions and dynamics and it may happen that glucose subtly exceeds the safe euglycaemic range without the patient's awareness [14].

To overcome SMBG limits, during the last 10–15 years, continuous glucose monitoring (CGM) sensors have been developed. The invasiveness of CGM sensors based on glucose-oxidase remains only minimal, but glucose concentration can be measured in real time with a 1–5 min sampling period and for up to 7 consecutive days (with the perspective of increasing the duration of their life up to 14 days in the next few years) [15–17]. One of the first proposed CGM sensors, which is still present in the market, is the microdialysis implementation proposed in Italy in the early 2000s by the Italian pharmaceutical company Menarini Diagnostics (Florence) [18]. In order to reduce patient discomfort, some research prototypes of non-invasive sensors (NI-CGM), not entering into direct contact with blood and based on e.g., dielectric properties measurable at the skin level, have been also proposed [19,20].

CGM time-series can be analyzed retrospectively to evaluate glucose variability, e.g., [21]. In clinical practice, it has already been demonstrated that the use of CGM sensors can significantly improve diabetes control and reduce HbA1c (a clinical indicator marking the quality of glycemic control and predictive of diabetes complications) [22–24], and CGM has been recommended, possibly integrated in the so called sensor-augmented pump, for the treatment of subjects prone to hypoglycemia, e.g., [25,26]. Langendam *et al.* [27] did a comprehensive review of the clinical investigations using CGM by elaborating more than 1,300 references. Telemedicine applications including the use of CGM and mobile phones have been also discussed e.g., in [28–31]. Apart from off-line analysis of quasi continuous glucose recordings, CGM sensors allow interesting online applications, as the generation of hypo and hyperglycemic alerts ahead in time, with the possibility for the patient of treating/mitigating the event timely (by a sugar ingestion to compensate an hypo or an insulin administration to tackle an hyper), see [32] for a review. Moreover, the CGM sensor is crucial in the development of the artificial pancreas (AP), *i.e.*, a minimally-invasive pump which subcutaneously administers insulin according to a temporal profile determined in real-time by a sophisticated closed-loop control algorithm that has CGM measurements as one of its key inputs, see e.g., [33–38]. However, for the inclusion of CGM sensors in the clinical routine, some issues have still to be dealt with, including reimbursement (provided in Italy, e.g., by only a few regional/local public health services) but also important technological problems related to accuracy and precision of the measurements. As a matter of fact, even if some commercial CGM sensors have received a positive evaluation from several governmental or independent authorities, such as the Food and Drug Administration (FDA) in the US, they cannot replace SMBG in clinical practice yet, and they can only complement it.

In this context, it is evident that the development of advanced technologies for the treatment of diabetes, universally accessible and easily usable from both the patients' and physicians' point of view, present several challenging aspects in different areas of scientific research, ranging from medicine to physics, electronics, chemistry, ergonomics, data analysis and signal processing, control engineering, software development, to mention but a few. The necessity of cross-fertilization among several research areas to achieve improvements in fighting diabetes and its complications is well established, as witnessed by the recent birth of several annual interdisciplinary conferences on diabetes technologies held in both the US, e.g., the Diabetes Technology Meeting (12th edition in 2012), and Europe, e.g., the Advanced Technologies and Treatments for Diabetes Conference (5th edition in 2012).

The present paper has the aim of reviewing Italian contributions to the development of CGM sensors for diabetes management. It is worth noting that, while it is one of the paper's aims to contextualize Italian contributions within the much wider context of the international research, making a comprehensive review of the CGM state-of-the-art is however outside the scope of the present work and of the Special Issue which contains it.

The outline of the paper is as follows: Section 2 will be devoted to the presentation of minimally-invasive CGM systems. After a short presentation of the international market scenario, we will describe the microdialysis implementations proposed by Menarini Diagnostics, partially developed in cooperation with the Tor Vergata University (Rome). Then, we will switch to the algorithm side and we will present the “smart CGM sensor” architecture developed by our group at the Department of Information Engineering at the University of Padova. Such an architecture conceptually consists in a cascade of a commercial CGM sensor and independent software modules, able to work in real-time and fed by the CGM output signals, the aim being to improve accuracy and precision of the sensor measurements, timeliness and effectiveness of patient management. Finally, we will discuss the use of CGM sensors in clinical trials performed in Italy, with special attention to those put into practice at the Department of Clinical and Experimental Medicine of the University of Padova—Medical School within projects recently supported by the European Commission under the 7th Framework Programme (FP). In Section 3, we will focus on NI-CGM. We will present the products proposed in the international research community, including some Italian contributions, both from industry (Pignolo SpA, Bergamo) and public institutions (ISIB-CNR Padova, Polytechnic of Bari). Then, challenges in building the models needed to reliably estimate glucose from the measured physical quantities will be discussed, making particular attention to a modeling approach pursued by our group. In Section 4 we will conclude the paper by briefly discussing possible further developments of CGM sensors and, in particular, some research lines active in Italy.

## 2. Minimally Invasive CGM Sensors

### 2.1. Sensor Technology and Manufacturers

Minimally invasive CGM sensors can essentially be divided into two categories: implantable needle-type enzyme sensors, and systems based on the use of a micro-dialysis probe coupled with a glucose biosensor. The first class includes e.g., the Guardian^®^ Real-Time (Medtronic MiniMed, Northridge, CA, USA), approved by FDA in 2005 [39], the Dexcom^®^ Seven^®^ and SevenPlus^®^ (Dexcom, San Diego, CA, USA), approved by FDA respectively in 2007 and 2009 [40] and the Free Style Navigator™, (Abbott Laboratories, Alameda, CA, USA), approved by the FDA in 2008 [41]. The most known CGM sensors that rely on microdialysis technology are the GlucoDay^®^ (Menarini Diagnostics, Florence, Italy), which received the Conformité Européenne (CE) mark in 2002 [42] and the SCGM 1 (Roche Diagnostics, Mannheim, Germany) [43]. Nice reviews of how the above cited sensors (but also others) work at the biochemestry level, with a critical discussion of pros, cons and perspectives and a comprehensive bibliography, are reported in [44–46], to which we refer the reader for details.

Briefly, needle-type enzyme sensors exploit glucose-oxidation reaction and measure the current flowing from the working to the counter electrode. The glucose-oxidase measurement principle is based on the generation of hydrogen peroxide via the enzyme glucose oxidase. After this step, a mediator conveys the electrons to the working electrode, where a potential is applied to oxidize the mediator itself. Since the sensor is implanted, enzyme and mediator should be immobilized onto the electrode surface to avoid them to dissolve in the interstitial fluid. A popular mediator is oxygen, because it is available in the interstitial fluid without requiring immobilization [45]. However, since oxygen concentration in interstitial fluid can be several hundred times smaller than the glucose concentration, techniques to limit glucose concentration should be adopted [47,48].

As far as microdialysis technology is concerned, the following brief description can be given. A hollow fiber, membrane permeable to glucose and other small molecules and impermeable to larger molecular species, is inserted subcutaneously. A fluid isotonic to the interstitial fluid, but containing no glucose, is pumped through the membrane fibers, so that the glucose in the interstitial fluid, driven by osmotic forces, diffuses through the membrane into the fluid stream, and the glucose concentration in the pumped fluid reaches an equilibrium with the glucose concentration in the interstitial fluid. The fluid flowing through the microdialysis membrane is then pumped to a glucose detector, which usually measure glucose with the amperometric approach, exploiting glucose oxidase and oxygen. The major advantages of microdialysis are the possibility of exposing the detector to atmospheric oxygen, (avoiding the deficit that characterizes glucose oxidase electrochemical sensors using O_2_/H_2_O_2_ as mediator), and the fact that the measurement is not affected by biofouling mechanisms, since the sensor is outside the body. However, new issues are represented by the necessity of a biocompatible microdialysis membrane, and by the time lag due to the pipe between the microdialysis membrane and the glucose sensor.

As better detailed in [18,44], the GlucoDay^®^ implementation of microdialysis technology for CGM was composed by a programmable microperistaltic pump, with a flow rate equals to 15–100 μL/min, a fluid line made of nylon in all the sections, apart from the peristaltic pump, a miniaturized wall jet flow cell (which contains the glucose biosensor, made of a platinum electrode and a three layers membrane system), an electronic circuit (with a low power microprocessor), a 9 V battery supply (for recording data continuously for 48 h), a fluidic pressure sensor, a microcontroller (for pump speed programming, signal acquisition and data storage), a disposal microdialysis probe, two plastic bags (one for the flowing buffer and the other to collect waste products), a display with a keyboard, and an interface for downloading the data to PC. The apparatus weights 245 g (without battery) and is contained in a pouch usually worn as a belt. The dialysis microfiber is inserted by the physician under the skin in the abdominal region of the patient. The glucose sensor consists of a platinum anode (diameter 0.4 mm) melted into a glass cylinder inserted into a silver tube that works as a cathode. The electrode is covered by three membranes: a cellulose acetate membrane, (which removes the electrochemical interference from ascorbic and uric acid, allowing the passage of hydrogen peroxide only), an enzymatic membrane, (where glucose oxidase is immobilized by cross linking with glutaraldehyde), and a polycarbonate membrane, (which is glucose limiting, in order to obtain a linear response to glucose), held in place by a small piece of Teflon tube of suitable diameter. The probes are constituted by 2 cm of hollow fiber (regenerated cellulose, with internal diameter of 0.17 mm and a molecular weight cut-off of approximately 15,000–20,000 Da), glued to two pieces of nylon tubing, and are sterilized with ethylene oxide gas. A pair of twisted tungsten wires are placed inside the fiber to prevent the collapse of the membrane.

The recently presented GlucoMen^®^Day is an evolution of the Glucoday^®^, produced by the same manufacturer. GlucoMen^®^Day is not CE certified yet. Part of its technical development and assessment has been made in collaboration with the Tor Vergata University in Rome. The manufacturer reports that GlucoMen^®^Day overcomes various shortcomings of its predecessor GlucoDay^®^ [49]. It is smaller and more compact, and the display and control unit have been separated from the actual monitoring device. In addition, preliminary evaluations report that it is more stable, has a longer lifetime and allows a monitoring period up to 100 h, minimizes bio- and electro-chemical interferences and embeds more effective algorithms for signal processing and data management. The new device exploits a planar electrode obtained by the screen-printed technology as electrochemical probe, coupled with a microdialysis fiber for continuous glucose monitoring [50]. Another major novelty is the chemical modification of the electrodes with Prussian Blue [51]. Experimental data *in vitro* showed that GlucoMen^®^Day is robust against the most common electrochemical interferences, suggesting that this device may become the CGM system of choice for those patients who require either regular administration of drugs or their glycemia to be tightly controlled in the intensive care unit [52].

### 2.2. Algorithms for CGM Sensors

Even if it has been recently demonstrated that the use of CGM sensors can improve glycemic control and reduce the occurrence of hypoglycemic and hyperglycemic events [23,24], the performance of these devices in terms of accuracy, measured e.g., by means of the diabetes-specific correlation analysis called “Clarke error grid” and its extensions [53,54], is still inferior to that of SMBG measurements and laboratory systems [14,27]. To improve the quality of CGM measurements several algorithms have been proposed, usually formulated by considering general signal processing aspects rather than possible sensor-dependant sources of error, e.g., related to specific sensor physics, chemistry and electronics. A quite detailed review of many of these algorithms can be found in [55,56].

**Figure 1.**
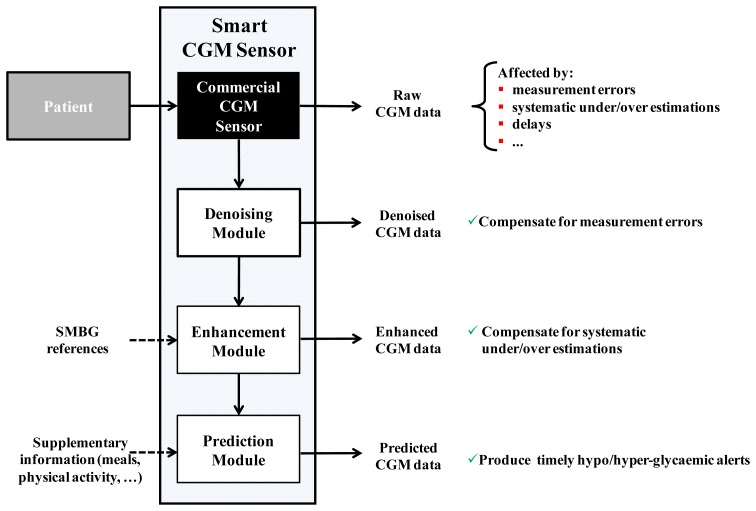
An example of the “smart” CGM sensor architecture: cascade of a commercial CGM sensor (black box) and three software modules working in real time for denoising, enhancement and prediction. The denoising module receives in input raw CGM data and returns a smoothed CGM time-series. The enhancement module processes the smoothed CGM data and produces a more accurate CGM profile. The prediction module forecasts the future glucose values, from which “preventive” alerts can be generated before critical hypoglycemic/hyperglycemic events occur.

In particular, our group proposed the concept of “smart” CGM sensor, consisting of a cascade of a commercial CGM sensor and several software modules dedicated to improve accuracy, precision and timeliness of glucose measurements. As illustrated in Figure 1, the datastream of glucose concentration readings produced in output by the sensor (any of the commercially available sensor could be conceptually considered) feeds a series of software modules, in this case three modules for denoising, enhancement and prediction, respectively.

**Figure 2.**
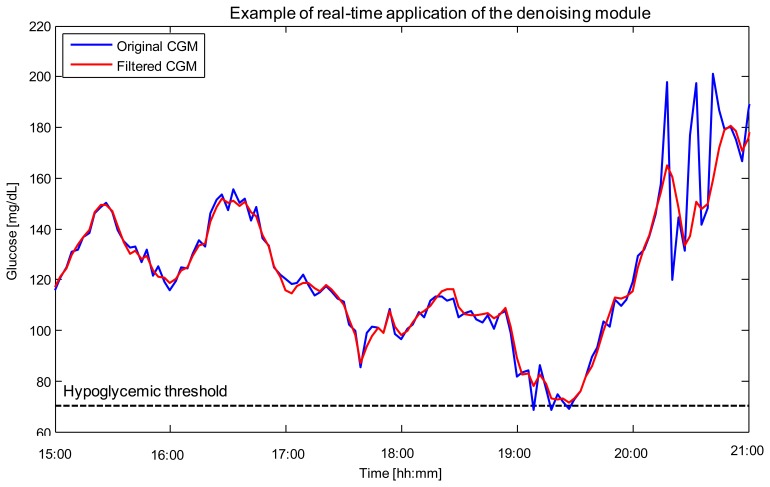
Denoising. Example of real-time application of the algorithm in [59] to a representative Glucoday^®^ time-series measured in a diabetic volunteer. Original (blue) and denoised (red) CGM profiles are shown. The black horizontal line represents the hypoglycaemic threshold.

Denoising is related to the uncertainty of CGM data, *i.e.*, glucose sensor readings are corrupted by random noise which complicates their interpretation and use. From a practical perspective, the greater the uncertainty, the higher the number of false hypoglycemic/hyperglycemic alerts potentially generated, with significant nuisance for the patient. Of note is that, in CGM, the signal-to-noise ratio (SNR) can vary among sensors, among individuals using the same sensor, and also during a given monitoring. However, the first approaches proposed in the literature usable for denoising, e.g., [57], were not able to cope with SNR variability. In 2010, our group proposed an on-line algorithm, implemented through the use of a Kalman Filter (KF), whose parameters could be individualized to the patient in a burn-in interval by an automatic procedure based on likelihood maximization [58]. The performance of such an approach was proven to be superior to that of the filters with fixed parameters incorporated within the firmware of commercial CGM sensors. In 2011, the algorithm was refined in order to cope, on-line, also with the variability of the SNR during the monitoring [59]. An example of use is shown in Figure 2 for a representative Glucoday^®^ time-series measured in a diabetic volunteer.

The CGM signal returned by the sensor (blue) is affected by noise with visibly time-varying variance. The denoised profile returned by the algorithm (red) presents reduced spurious oscillations which could generate false hypoglycemic/hyperglycemic alerts (e.g., around time 19:10). Notably, the ability to track SNR variability allows the algorithm to reduce the noise affecting the signal in the interval 20:00–21:00 without oversmoothing the signal in intervals where the SNR is more favourable, e.g., in the interval 15:00–17:00.

**Figure 3.**
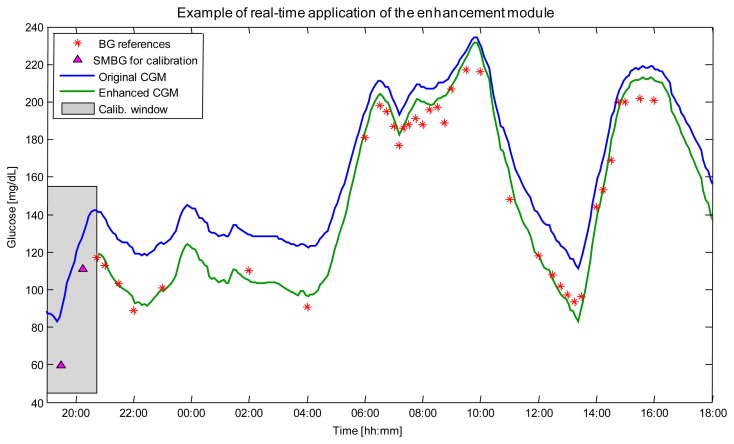
Enhancement. Example of real-time application of the algorithm in [66] to a representative DexCom^®^ SevenPlus^®^ time-series measured in a diabetic volunteer. The gray rectangle highlights the two SMBG values (magenta triangles) and the portion of CGM data (blue line) used by the algorithm to estimate the internal regressor parameters. Original CGM data (blue line) and enhanced glucose profile (green line) are then shown against BG references (red stars), collected in parallel using a laboratory instrument.

The enhancement block in Figure 1 deals with the problem of accuracy. CGM sensors measure glucose in the interstitial fluid of the subcutis. Since the existence of a blood-to-interstitium glucose kinetic (BG-to-IG), CGM time-series result always affected by a distortion when they are compared to “gold standard” blood glucose (BG) concentration references measured by laboratory instruments. The most visible effect of the distortion induced by BG-to-IG kinetics is a temporal delay, which further increases the delay of CGM due to the processing times required within the sensor [60]. Another important source of systematic under/overestimations has connection with calibration issues [61]. The problem of accuracy is well described by the example of Figure 3, relative to an experiment where a DexCom^®^ SevenPlus^®^ CGM profile (blue line) was measured in parallel to BG references (red asterisks) assessed using a gold standard laboratory instrument. Systematic differences between CGM signal and BG references (of the order of 20% in the interval 22h–04h) cannot be explained by the unavoidable distortion due to BG-to-IG kinetics and are likely due to calibration problems. Inaccuracy can become extremely critical especially during the night when the patient is asleep and, for instance, a systematic overestimation of glucose levels could expose him/her to risky situations. To deal with the accuracy problem, several approaches (often working only off-line) have been proposed in the literature, e.g., by King *et al.* [62], Knobbe and Buckingham [63], and Barceló-Rico *et al.* [64]. These approaches correct the CGM signal by exploiting the SMBG samples sporadically measured by the patient. In 2010, our group proposed an on-line enhancement method exploiting an Extended Kalman Filter (EKF) [65]. The model beyond the EKF accounted for errors due to calibration, sensor drifts due to loss of sensitivity, and BG-to-IG kinetics. Since nonlinearities of EKF rendered its implementation cumbersome, in 2012 the same idea for CGM signal enhancement was developed within a simpler procedure [66]. Briefly, as two suitably collected SMBG values are available, a portion of CGM data in correspondence to them is selected and a nonparametric deconvolution procedure is applied. Then, the parameters of a linear regressor are estimated and applied to correct the forthcoming CGM values. An example of the application of the algorithm of [66] on the representative dataset is displayed in Figure 3. The algorithm used the two SMBG values (magenta triangles) to estimate the parameters of the regression model which is then used to correct the original CGM data (blue line) and provides an enhanced CGM profile (green line). The comparison with the BG gold standard references collected in parallel (red stars, not used in the enhancement procedure) shows that most of the systematic under/overestimations not explainable by BG-to-IG kinetics were removed and a much more accurate CGM profile was provided for the management of the individual therapy.

The last block in the scheme of Figure 1 deals with prediction. In a proof-of-concept study published in 2007 [67], our group demonstrated, by using two simple prediction algorithms based on polynomial and autoregressive models of order 1, that generating hypo/hyperglycemic alerts is possible ∼20 min ahead of time with respect to the forthcoming event by resorting to short-term glucose prediction strategies. This margin of time may allow to prevent e.g., hypoglycemia, since it is comparable, if not greater, than the interval required for an oral glucose intake to reach the blood circulation [32]. Since then, many research groups worldwide developed several prediction approaches based on e.g., autoregressive models of high order [68], recursive linear models and change detection algorithms [69], neural networks/machine learning [70–72] and even combination of approaches [73,74]. An open issue concerning prediction algorithms is that all of them require to tune one or, often more, parameters (e.g., a forgetting factor allowing to weigh past data in different way, the prediction horizon, model order). An index to optimize parameters of prediction algorithms was proposed by Facchinetti *et al.* [75]. At the same time, modifications of the Clarke error grid analysis have been suggested to assess the accuracy of prediction algorithms [76,77]. Present research of our group at the University of Padova concerns the design of predictive algorithms based on neural networks (NNs), developing further the approach originally presented in Perez-Gandia *et al.* [70] in order to exploit, in addition to CGM, supplementary information, e.g., the amount of carbohydrates ingested by the patient.

**Figure 4.**
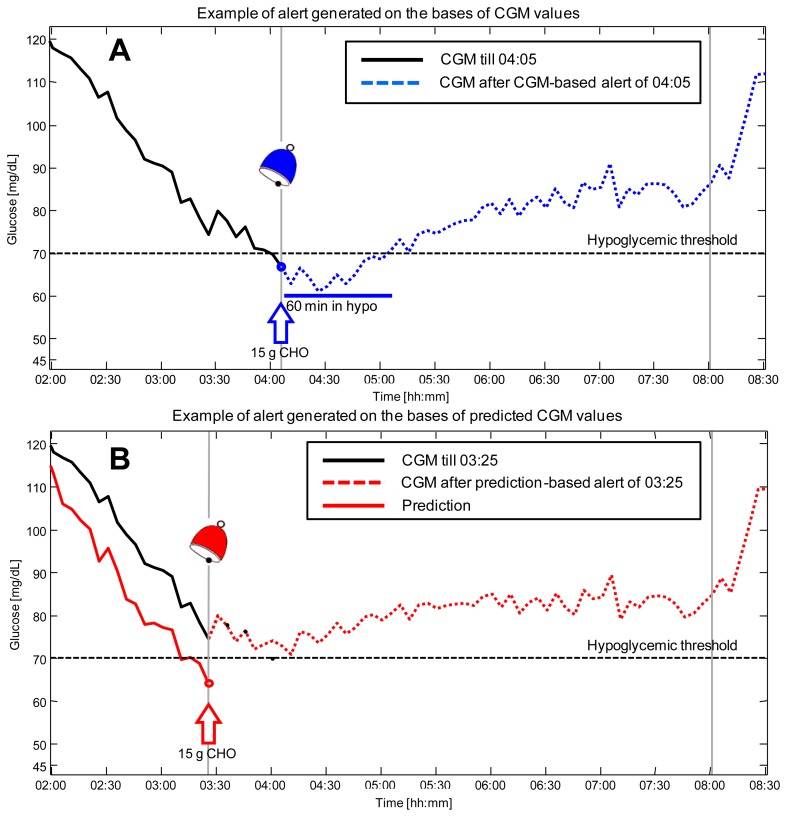
Prediction for alert generation (simulation context). (**A**). Hypoglycemic alert generated on the basis of CGM data. CGM data before (black line) and after (blue dashed line) the carbohydrates ingestion (blue arrow) in response to alert (blue bell) generated at hypoglycemic threshold crossing. (**B**). Hypoglycemic alert generated on the basis of predicted CGM data. CGM data before (black line) and after (red dashed line) the carbohydrates ingestion (red arrow) in response to alert (red bell) generated when predicted CGM (red line) exceeds hypoglycemic threshold.

Figure 4 shows the results obtained in simulation by employing the recently published approach, a NN based algorithm which exploits information on meals thanks to the incorporation of a suitable mathematical model of glucose absorption from the stomach [78]. Panel A shows the scenario in which the hypoglycemic alert is generated on the basis of CGM data, *i.e.*, the alert is given at 04:05 (blue bell) when the CGM profile (black line) exceeds the hypo threshold (black dotted horizontal line). The glucose concentration in the following hours, after 15 g of carbohydrates are ingested by the patient, is reported (blue dashed line). As visible, the patient spends 60 minutes in the critical hypoglycemic range. Panel B shows what happens if the predicted profile obtained by the NN algorithm (with a prediction horizon of 30 min) is exploited in real-time to generate hypoglycemic alerts. In this second scenario, the predicted glucose profile (red line) is forecasted to cross the hypoglycemic threshold at time 03:25. This results in the generation of a preventive alert (red bell) and in an advice suggesting the patient to ingest 15 g of carbohydrates. Thanks to the generation of the alert ahead of time and the timeliness countermeasure taken by the patient, glucose concentration in the following hours (red dashed line) never exceeds the hypoglycemic range.

To conclude, the smart sensor algorithms in Figure 1 have been ordered to maximize the global utility of the system. In fact, before compensating for a lack of accuracy by using the enhancement module, it is beneficial to improve the SNR by denoising. Similarly, prediction of future glucose levels to obtain anticipation in alert generation is more precise and effective if CGM data are first smoothed and enhanced. However, an advantage of the “smart” CGM sensor architecture is that all the modules are mutually independent, *i.e.*, if one module is removed the others can still work. Another advantage is that all modules work in cascade to any CGM sensor, independently from the CGM manufacturer. Recently, the smart sensor architecture of Figure 1 was successfully applied in a clinical study involving 24 diabetic subjects [79].

### 2.3. Clinical Tests of Minimally-Invasive CGM Sensors

The literature on clinical applications of minimally-invasive CGM is extremely wide and, at the time of writing, more than 2,000 papers on the topic are indexed in the PubMed bibliographic system [80]. While the interested reader may refer to the articles already cited in the Introduction of the present paper for a more comprehensive overview of the state-of-the-art, in this subsection we intentionally limit ourselves to describe some of the research progresses obtained in Italy in the last decade.

The first use of CGM recordings in clinical investigations was for retrospective analysis. In particular, to the best of our knowledge, the first Italian experience with CGM sensors was carried out with the Menarini GlucoDay^®^ microdialysis system in a multicenter study coordinated by the University of Padova which involved 70 diabetic patients from several different Italian clinics [81]. Results demonstrated a good correlation of CGM with gold standard BG references over a wide range of levels (40–400 mg/dL), with 97% of the measurements falling in the two most favourable regions of the so-called Clarke error grid (see Figure 6 in [81]) and an average percentage error of −2.0% in the hypoglycemic range (<70 mg/dL), 6.9% in the euglycemic range (70–180 mg/dL), and 11.2% in the hyperglycemic range (>180 mg/dL). In studies later conducted with the same sensor at the University of Perugia [82], similar performance was observed during insulin-induced hypoglycemia and subsequent phases. In [83], frequency and duration of nocturnal hypoglycaemic events were studied in 57 diabetic subjects under insulin therapy and a strong association between bedtime glucose and occurrence and duration of nocturnal hypoglycaemia was found. In a recent study performed at the San Raffaele Hospital (Milan), similar results were found in a group of adolescents with poor metabolic control [84]. In Maran *et al.* [85] the effects of exercise on subsequent hypoglycaemia were investigated by the GlucoDay^®^ in 8 well-controlled diabetic patients with 30 min of both intermittent high-intensity exercise and moderate-intensity exercise in random order. In [86], glucose variability indexes obtained from maternal CGM profiles measured by the GlucoDay^®^ were correlated with fetal growth parameters for 80 pregnant women studied in the diabetes clinics of Padova, Pisa, and Florence. By using the Medtronic-Minimed sensor in 17 diabetic patients, the Ravenna Hospital group demonstrated that insulin aspart formulation has a better effect on glucose variability than lispro insulin formulation [87]. Finally, in the last months, other Italian clinical research groups (Tor Vergata-Rome, Naples, Padova) have focused the attention on the effect of glucose variability as measured by retrospective CGM on cardiovascular risk and oxidative stress [88,89] suggesting new indexes of hyperglycaemia other than Hba1c as an indicator of intermediate glucose control [90].

The use of CGM sensors in real time was tested in trials performed at the Department of Clinical and Experimental Medicine of the University of Padova within two projects funded by the European Commission under FP7, Diadvisor (2008–2012) and AP at home (2010–2014).

The Diadvisor project [91] aims at developing a unique tool, called DIAdvisor™, to help diabetic patients in the management of insulin therapy. By a prediction of glucose excursions, DIAdvisor™ is expected to warn the patient online about the risk of moving out of aimed glucose range. Moreover, the activation of an advisor component is scheduled to offer solutions to stay in a safe glucose range. In a first trial, insulin delivery, meal intakes, CGM data as well as vital sign parameters such as heart and respiratory rates were collected in 90 patients recruited in the three clinical centres participating to the project, *i.e.*, Montpellier (France), Padova and Prague (Czech Republic). Each patient was monitored for three days under standardized conditions in the hospital (with plasma insulin and gold standard blood glucose samples frequently collected in parallel by laboratory instruments) and seven days at home. These data allowed to the development of prediction and advisor algorithms that were installed on an ultra-mobile PC with a graphical patient interface and a wireless connection to a DexCom Seven Plus CGM sensor. In a second trial of the project, the DIAdvisor™ prototype was tested on 29 diabetic volunteers. Results showed that DIAdvisor™ is able to provide successful prediction of blood glucose at a 20-min horizon and coherent advices for treatment correction in diabetic patients at rest and during challenging conditions in a standardized hospital environment [92].

The aim of the AP at home project [93] is to develop a subcutaneous-subcutaneous AP, *i.e.*, glucose sensing and insulin infusion occur both in the subcutaneous tissue, usable under daily life conditions. In the project, two different approaches are being considered: a two-port AP system and a pioneeristic single-port AP system. The two-port AP system will use off-the-shelf-components for the glucose sensor and the insulin pump in combination with two different candidate closed-loop control algorithms developed by the University of Pavia (Italy) [94] and the Cambridge University (UK) [34]. In the single-port AP system, continuous glucose monitoring and insulin infusion will take place via a single catheter [95]. The first clinical trial (performed in 2011) investigated the performance of the closed-loop *vs.* open-loop control algorithms in 47 patients recruited in the six clinical centres participating to the project, *i.e.*, Amsterdam (The Netherlands), Cambridge, Graz (Austria), Montpellier, Neuss (Germany), Padova in a randomized study during three clinical admissions (including three meals and an exercise bout). CGM data were collected by the Dexcom Seven Plus sensor. Preliminary results showed the feasibility and superiority of automated management of the closed-loop systems [96].

## 3. Non Invasive CGM Sensors (NI-CGM)

### 3.1. Sensor Technology and Manufacturers

The term non-invasive CGM (NI-CGM) conventionally refers to technologies able to measure glucose levels without entering into direct contact with the blood. In other words, no skin penetration by needles is required, as for the minimally-invasive sensors described in Section 2. These technologies usually exploit optical, thermal, acoustic, electromagnetic or electric principles (or a combination of them) and require suitable mathematical models to infer on glucose concentration in the blood from the available physical measurements, normally obtained at the level of skin and underlying tissues. NI-CGM is appealing for obvious reasons related to patient comfort, but largely challenging because of many potential environmental and physiological interferences. As a matter of fact, several of the NI-CGM devices proposed in the literature have been abandoned or are still at the prototype level. In the following, only the most known NI-CGM approaches are illustrated and only briefly, the aim being contextualizing the Italian contributions to the field, both from industry and academia. For a more comprehensive review of NI-CGM technologies, we refer the reader to [19,20].

Optical NI-CGM techniques are based on a beam of light shot at the skin and on the measurement of the reflected, scattered and absorbed components. For example: TANGEST [97] and MedOptix™ [98] devices employ Near InfraRed spectroscopy (NIR) wavelengths in the range of 750–2,000 nm [99] to measure absorption coefficients modulated by glucose concentration changes; the system by Shen *et al.* [100] employs a different waveform length range (2,500–10,000 nm), referred to as Mid InfraRed spectroscopy (MIR), which exhibits less scattering phenomena and more absorption than NIR; C8 MediSensors optical glucose monitor™ [101] exploits Raman spectroscopy (this device has applied for the CE mark); the OrSense NBM-200G [102] implements occlusion spectroscopy by exploiting the property of glucose to decrease the diffusion coefficient and the enhanced transmission of light due to erythrocyte aggregation that can be induced *in vivo* by applying, for a couple of seconds, a pressure greater that the systolic one to a fingertip through a ring-shaped sensor probe (this sensor received the CE mark in 2007); the GlucoLight Sentris-100™ resorts to optical coherence tomography [103]. Proposals of measuring glucose concentrations from the eye using fluorescence technology (together with contact lens and hand-held external light source/detector) or polarimetry have been also presented, see e.g., [104,105]. An Italian contribution in this field was provided by the Glycolaser^®^, a light-weight portable device for the assessment of glycaemia developed by Pignolo SpA (Bergamo, Italy) which, according to the (small) amount of information reported in the patents [106,107], exploits light in the frequency range between 500 and 1,000 nm.

NI-CGM thermal technologies include the system presented in [108] to measure, at the skin level, the IR signals naturally emitted from the human body as a result of glucose concentration changes.

Acoustic NI-CGM technologies such as the Aprise™ by Glucon [109] exploit the property of blood and tissues to generate ultrasound waves (that can be measured by a microphone) when they are illuminated by laser pulses at specific wavelengths.

Electromagnetic techniques measure dielectric parameters of blood that are modulated by glucose level changes, exploiting the electromagnetic coupling between two inductors turned around the blood sample [110]. In Italy, the research group at ISIB-CNR (Institute of Biomedical Engineering of the Italian National Research Council) of Padova recently investigated the feasibility of such a kind of sensing approach and a prototype, developed in partnership with a private company (Bellco SRL, Mirandola, Italy), was successfully tested *in vitro* for sensing glucose concentrations in different solutions with similarities with blood [111].

Finally, electric technologies, often referred to as impedance spectroscopy (IS) or dielectric spectroscopy (DS) allow the investigation of the dielectric properties of a tissue based on the measure of the tissue impedance applying a current of known intensity. The application of alternating current at different frequencies allows the measurement of the impedance as a function of the frequency, obtaining the so-called dielectric spectrum. An example of use of this approach is the Pendra [112], a device which received the CE mark in 2003 but abandoned in 2005 when serious issues concerning its accuracy were raised as result of post-marketing reliability studies [113]. In Italy, the ISIB-CNR in Padova developed a prototype for *in vitro* monitoring of glucose concentrations in different solutions [114]. More recently, a research group of the Bari Polytechnic presented a prototype sensor for NI-CGM based on DS featuring capacitive fringing field electrodes working in a frequency range between 1–160 MHz [115].

Rather than focusing on a single technology, some NI-CGM devices resorted to a combination of approaches and are thus referred to as multi-sensors. Among them, the system developed by a Swiss company named Solianis Monitoring AG [116], whose IP and technology is now held by Biovotion AG (Zurich, Switzerland). This device embeds into the substrate in contact with the skin several sensors for the bio-physical characterization of the skin and underlying tissues. In particular, glucose related signals are obtained from DS fringing field capacitive electrodes, while environmental and physiological processes that can interfere with the measurements of the main glucose signals are measured with temperature, optical, humidity accelerometer and additional DS sensors. A different, yet recent, multi-sensor approach is that of the GlucoTrack [117], which exploits a mix of thermal, acoustic, and electromagnetic technologies. Finally, a prototype has been developed by Amaral and co-workers [118] which employ a combination of DS and MIR based sensors.

Concluding, it is worth mentioning that the present review is far from being exhaustive. For instance, we have deliberately omitted to report techniques which cannot properly classified as non-invasive, e.g., techniques involving the creation of micro-pores such as the SpectRx Inc. [119], or based on reverse iontophoresis such as the GlucoWatch^®^ by Cygnus Inc. [120], (approved by FDA in 2002, but withdrawn from the market in 2007) and sonophoresis (e.g., the Symphony™ by Echo Therapeutics [121]), or the exploitation of micro-needles. We refer to e.g., [19,20,122,123] for more information on these technologies. Moreover, it is also worth mentioning that other techniques exploiting biological fluids (such as saliva, urine, sweat and tears) other than the plasma have been also considered in the literature, mainly for intermittent glucose monitoring [124].

### 3.2. Algorithms for NI-CGM Sensors

In order to estimate glucose levels from the measurements obtained using the aforementioned technologies, an algorithm, possibly relying on a formal mathematical model, is needed. Robust mechanistic/physical descriptions linking glucose variations with changes in the parameters of the skin and underlying tissues are in general unavailable and only some attempts to build physical models have been documented in the literature [101,117,125]. Thus a black-box strategy is normally used, where the inputs are the measurements provided by the sensor and the output is glucose concentration. As a general remark, a global (“population”) model valid for all subjects would be more appealing in practice, since no studies at the individual patient level would be needed to use the NI-CGM device.

**Figure 5.**
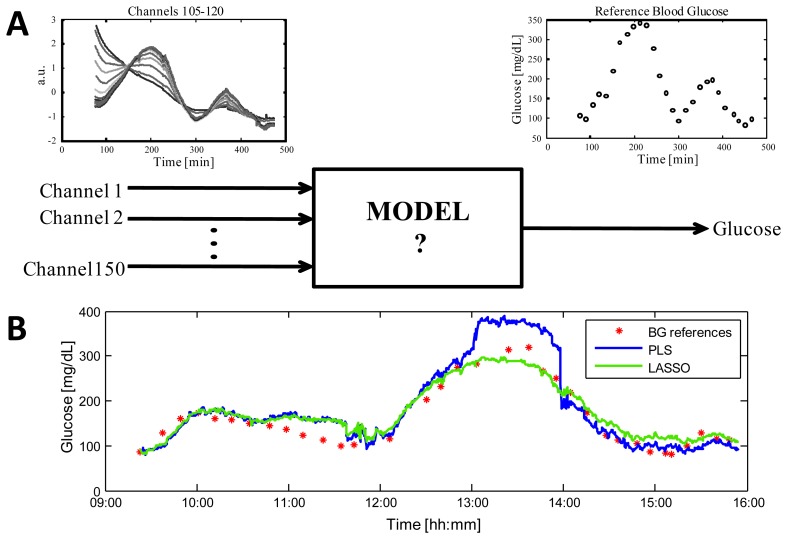
(**A**) Identification of the model allowing to estimate glucose concentration from the 150 signals returned by the Solianis Monitoring AG Multisensor system. The inlaid graphs show, for a real subject, the time-course for the 16 representative channels numbered as 105–120 (left) and the reference BG glucose concentration levels measured in parallel (open bullets, right). (**B**) Example of estimated glucose profiles during model test for a representative experimental session with the PLS (blue line) and with the LASSO (green line) models against BG samples [129].

Since many of the techniques mentioned in Section 3.1 provide a set of correlated measurements (e.g., spectroscopy sensors), or involve the use of different technologies with a high number of measured signals (e.g., multi-sensors), a multivariate regression model is the most natural candidate for NI-CGM [126,127]. Figure 5 shows the key aspects of the problem, considering as an example, data from the Solianis Monitoring AG Multisensor. In particular, panel (A) shows the time-course of 16 representative channels (left) taken from the 150 measured by the Multisensor in a real subject and the time-series of the reference BG glucose concentrations measured in parallel by a gold standard technique as reference (right).

The unknown model (middle) must allow estimation of glucose concentrations from the physical quantities measured by the sensor. Having fixed model structure, e.g., linear, the problem of identifying the unknown model parameters is however usually ill-conditioned and numerical issues also arise from the non-orthogonality of the subset of predictors and from the potentially high dimension of the measurement space. In order to overcome to these issues, only identification methods controlling the model complexity should be considered, also for avoiding overfitting that could deteriorate accuracy of estimated glucose profile during model test, *i.e.*, when glucose is estimated with data not used during the model identification stage. A popular method usable for such a scope is Partial Least Squares (PLS), see [127,128] for applications in the identification of multivariate linear regression models for NI-CGM. Our group has recently shown that the Least Absolute Shrinkage and Selection Operator (LASSO) outperforms PLS when there is the need of merging data from different sensors in order to compensate environmental and physiological processes deteriorating the signals which mainly reflect glucose [129]. In particular, it has been shown that, for the multi-sensor platform of Solianis Monitoring AG, the model identified on training data by LASSO is more robust than that obtained by PLS when, on validation data, occasional spikes and jumps occur in some of the multisensor signals (as it may happen during the every-day use). Figure 5(B) displays the clear superiority, in terms in accuracy, of the profile obtained by the LASSO model over that obtained by the PLS model in a representative experimental session (see [129] for full results over 45 experimental sessions). It is possible to speculate that this improvement should also hold for other devices equally sensitive to endogenous and/or exogenous processes deteriorating the sensor signals mainly related to glucose.

### 3.3. Clinical Tests of NI-CGM Sensors

Outside the finely controlled conditions of laboratories or hospitals, NI-CGM is particularly challenging because of several sources of interferences, e.g., temperature, sweat, moisture, typical of daily-life. As a matter of fact, the several clinical trials conducted in the last years to assess NI-CGM devices provided several insights into the dynamical/physiological processes that could influence the tracking of glucose. For example, the Solianis Monitoring Multisensor was tested at the Zurich University Hospital in six type 1 diabetic patients undergoing a glucose clamp experiment, *i.e.*, glucose was forced to vary according to a predetermined profile, in order to test accuracy in discriminating different glucose levels and trends but also possible effects of electrolyte accumulation after repeated hypo and hyper excursions [130,131]. As far as other devices are concerned, Chen *et al.* reported a study with three prediabetic and 15 diabetic subjects using the TANGEST [132]. The GlucoTrack was tested during clinical trials involving 27 type 1 and 98 type 2 diabetic subject plus additional 10 healthy subjects [133]. The Or-sense device NBM-200G was tested during clinical trials with 12 type 1 and 11 type 2 diabetic subjects [134], the Aprise™ from Glucon Medical Ltd. was assessed during a study with 23 type 1 and 39 type 2 patients [109]. The C8 MediSensors optical glucose monitor™, according to the company website [135], is undergoing clinical trials for “demonstrating continued accuracy improvement”.

As far as Italian NI-CGM prototypes are concerned, to the best of our knowledge, only *in vitro* studies have been performed so far on the ISIB-CNR (Padova) systems described in [19,122]. The Glycolaser^®^ device (Pignolo SpA) was tested in 31 healthy and 136 diabetic subjects [136]. Investigations are reported to be underway for the device of the Polytechnic of Bari [115].

To conclude, it is worth remarking that, although the performance of the NI-CGM systems proposed so far never reached that of enzyme-based needle glucose sensors, research is very active. At present time, glucose trends are quite satisfactorily estimated. Considering the non-invasive nature of the presented technologies, this result can have an immediate impact in the current clinical practice, e.g., to integrate sparse SMBG data with an indication of the glucose trend to aid the diabetic patient in dealing with, in the short time scale, critical events such as hypoglycaemia [129].

## 4. Conclusions

CGM sensors are new emerging technologies that, as recently demonstrated in clinical studies, have the potential of significantly improving diabetes therapy. Retrospectively, the great amount of individual CGM data can be useful to better tune individual therapeutic plans and can also allow insightful assessments and developments of the concept of glycemic variability (relative to long, medium and short dynamics). In real time, CGM sensors can allow the development of new strategies for the treatment of diabetes, in both open-loop (alert generators) and closed-loop (AP). This explains why the potential market of CGM sensors is extremely broad (only in the US, estimated in hundreds of thousands of units per year) and several methodologies developed in academia can be of interest for industries and start-up companies.

To the best of our knowledge, at present time, at least four manufacturers of minimally-invasive CGM sensors (Abbott Laboratories, Dexcom, Medtronic Minimed, Menarini Diagnostics) and one manufacturer of NI-CGM sensors (OrSense) are present worldwide in the commercial market.

As documented in the present paper, Italian contributions to the field of CGM sensors in the last 10–15 years are remarkable. Menarini Diagnostics developed the first system utilizing microdialysis which obtained the CE mark in 2002 and recently presented an updated version of the system created by taking advantage from a collaboration with the Tor Vergata University in Rome. Our group at the University of Padova has proposed several algorithms to improve the accuracy and precision of CGM sensors and recently participated to clinical trials, involving the use of real-time CGM sensors applications, successfully performed under the aegis of projects supported by EU under FP7. As far as the challenging NI-CGM problem is concerned, interesting hardware prototypes have been proposed by small private companies (Pignolo SpA) as well by public research groups (ISIB-CNR Padova and Bari Polytechnic), but also models to reconstruct glucose concentration from the measured physical quantities have been proposed (our group).

Present research of our group at the University of Padova includes the development/refinement of algorithms for the smart sensor concept. In particular, we are investigating algorithms based on a new measure of risk [137] which explicitly considers the rate of change of glucose as a threat factor for the patient (e.g., risk levels in hypoglycemia and hyperglycemia are amplified in the presence of a decreasing and increasing glucose trend, respectively). As far as prediction algorithms are concerned, we are investigating how CGM information can be complemented by some other sources of information (composition of ingested meals, times and dosages of insulin administrations, amount of physical activity performed by the patient) in order to improve the ability to forecast hypo/hyperglycemic events, in terms of sensitivity, specificity and timeliness [138]. Another important open point concerns with transient artifacts due to sensor failures, which, in the case of minimally-invasive needle sensors, can be induced by, e.g., pressures applied to the sensor, patient movements, changes in the oxygenation of the sensor. These failures can be particularly critical in real-time situations, e.g., a systematic overestimation of glucose concentration during the night can expose the patient to severe health risk. Preliminary results obtained by a state-space modeling approach have been presented in [139]. Finally, further work is ongoing for improving the identification of models for NI-CGM by using techniques such as the Elastic-Net regression [140] and *ad hoc* calibration strategies. The effect of physiological disturbances, such as sweating, on the model identification procedure is also under assessment. From the clinical side point of view, ongoing studies on the AP will permit a better tuning of the algorithms in order to achieve increased percentage of time in glucose target and lower mean glucose levels while keeping the reduction of hypoglycaemic events. As shown from some preliminary results [141], this will allow the test of a wearable AP outside the hospital in real-life conditions.

## List of Abbreviations

CGM: Continuous Glucose Monitoring
AP: Artificial Pancreas
EU: European Union
SMBG: Self Monitoring Blood Glucose
HbA1c: Glycosylated Hemoglobin
CE: Conformité Européenne
NI-CGM: Non-Invasive Continuous Glucose Monitoring
FDA: Food and Drug Administration
7FP: 7th Framework Programme
SNR: Signal-to-noise Ratio
(E)KF: (Extended) Kalman Filter
BG-to-IG: Blood-to-Interstitium Glucose
BG: Blood Glucose
NN: Neural Network
AP: Artificial Pancreas
NIR: Near InfraRed
MIR: Mid InfraRed
IS: Impedance Spectroscopy
DS: Dielectric Spectroscopy
IP: Intellectual Property
PLS: Partial Least Squares
LASSO: Least Absolute Shrinkage and Selection Operator
